# Efficacy of pancreatic enzyme replacement therapy (PERT) in the treatment of gastrointestinal symptoms after gastric cancer surgery: study protocol for a multicenter randomized controlled open-label trial

**DOI:** 10.3389/fphar.2026.1779727

**Published:** 2026-03-11

**Authors:** Shuomeng Xiao, Jun Bu, Yingxin Wu, Yaoheng Lu, Peng Zhang, Kuan Liu, Yulong Zhou, Peng Liu, Fazhi Zhao, Chao Yang, Da Zhou, Qingchuan Chen, Weiyang He, Zhi Ding, Xiaodong Chen, Xiang Zhou, Ping Zhao, Huali Zhou, Rui Xu

**Affiliations:** 1 Department of Gastric Surgery, Sichuan Clinical Research Center for Cancer, Sichuan Cancer Hospital & Institute, Sichuan Cancer Center, Affiliated Cancer Hospital of University of Electronic Science and Technology of China, Chengdu, China; 2 Department of Gastrointestinal Surgery, Chengdu Second People’s Hospital, Chengdu, China; 3 Department of Gastric Surgery, Chengdu Third People’s Hospital, Chengdu, China; 4 Department of Gastrointestinal Surgery, Chengdu First People’s Hospital, Chengdu, China; 5 Department of Gastrointestinal Surgery, Mianyang Third People’s Hospital, Mianyang, China; 6 Department of Gastrointestinal Surgery, Mianyang People’s Hospital, Mianyang, China; 7 Department of Gastrointestinal Surgery, Langzhong People’s Hospital, Langzhong, China

**Keywords:** gastric cancer, gastrointestinal symptoms, pancreatic enzyme replacement therapy, pancreatic exocrine insufficiency, surgery

## Abstract

**Introduction:**

Pancreatic exocrine insufficiency (PEI), presenting as bloating or diarrhea, affects 26%–100% of patients after gastrectomy, delaying recovery and impairing quality of life. Pancreatic enzyme replacement therapy (PERT) is the standard PEI management, yet its efficacy after gastric cancer surgery remains controversial. This study evaluates PERT’s effectiveness in alleviating postoperative gastrointestinal symptoms.

**Methods and Analysis:**

In this multicenter randomized controlled trial, 204 patients undergoing radical gastrectomy will be randomized 1:1 to PERT or control. The PERT group will receive Pancreatin Enteric-coated Capsules (containing 450 mg pancrelipase microgranules) three times daily with meals for 1 month post-discharge. The primary outcome is the incidence of gastrointestinal symptoms at 1 month post-surgery, assessed using the MD Anderson Symptom Inventory Gastrointestinal Cancer Module (MDASI-GI). Secondary outcomes include nutritional status and treatment compliance.

**Discussion:**

This protocol establishes a framework for definitively evaluating PERT in gastric cancer patients. Results will inform evidence-based PEI management, with significant implications for survivorship care. By integrating patient-reported outcomes and nutritional metrics, we aim to validate a patient-centered strategy optimizing functional recovery after curative resection.

**Clinical Trial Registration:**

This study was registered with the Chinese Clinical Trial Registry (ChiCTR; registry number: ChiCTR2500102557). Date of registration: 16 May 2025.

## 1 Introduction

Gastric cancer ranks as the fifth leading cancer for both mortality and morbidity worldwide (Sundar et al., 2025). In 2022, approximately 360,000 patients were diagnosed with gastric cancer and 260,000 died from the disease in China (Zheng et al., 2024). Gastrectomy and D2 lymphadenectomy still play a crucial role in the treatment of gastric cancer. However, more than half of the patients suffered from gastrointestinal symptoms after surgery (Xu et al., 2022), such as abdominal distention, pain, diarrhea, which delayed patient recovery and affected quality of life.

Certain postoperative gastrointestinal symptoms are caused by pancreatic exocrine insufficiency (PEI). Clinical symptoms of PEI include steatorrhea, weight loss, bloating and malnutrition (Whitcomb et al., 2023). PEI is characterized by deficient enzyme secretion, leading to maldigestion, malabsorption, and malnutrition (Ghodeif, 2023; Whitcomb et al., 2023). Some studies showed that the incidence of PEI following gastrectomy was 26%–100% (St et al., 2017; Heneghan et al., 2015), which may be related to the absence of neural gastric reflexes, bacterial overgrowth, extensive denervation of the pancreas and asynchrony between delivery of the pancreatic enzymes and the food particles (Iivonen et al., 1998; MacGregor et al., 1977; Domínguez-Muñoz, 2009; Leth et al., 1991). Oral pancreatic enzyme replacement therapy (PERT) is the mainstay of treatment for PEI. Few studies have addressed PERT following gastrectomy for gastric cancer. Some studies founded that patients with gastrectomy got the symptomatic benefit of PERT, a positive response was seen in 78.9% (71.9%–85.2%) (Guman et al., 2022; Moore et al., 2023; Veeralakshmanan et al., 2020). However, another study considered that PERT had no effect on steatorrhea, bowel movements and observation time were only 14 days (Brägelmann et al., 1999). To date, there is no consensus on the use of pancreatic enzyme replacement therapy for gastric cancer after surgery and whether pancreatic enzyme replacement therapy can alleviate postoperative gastrointestinal symptoms is still unclear.

In this multicenter randomized controlled trial, we aim to evaluate efficacy of pancreatic enzyme replacement therapy (PERT) in the treatment of gastrointestinal symptoms after gastric cancer surgery.

## 2 Methods and methods

### 2.1 Trial design

**FIGURE 1:**
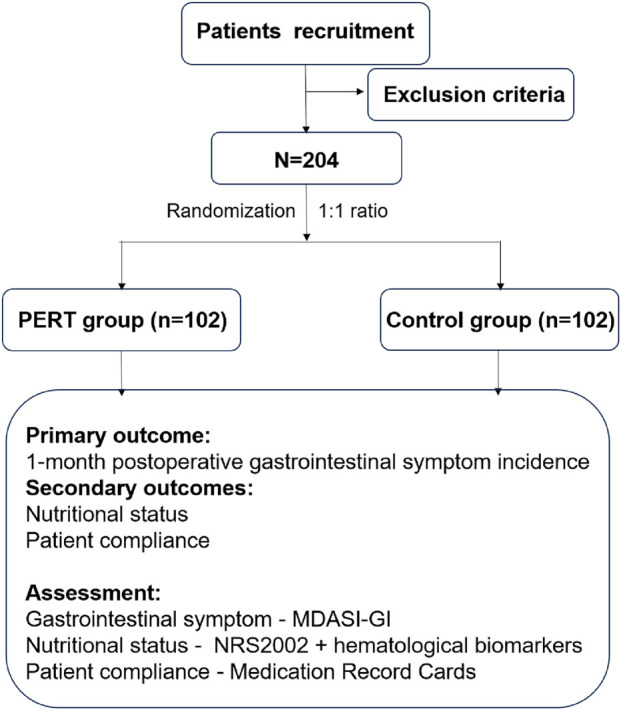
Flowchart of the study.

The PERT-GC01 trial is a multicenter randomized controlled trial (ChiCTR Identifier: ChiCTR2500102557), gastric cancer following radical gastrectomy will be randomized in a 1:1 ratio to the PERT group or the control group. A randomization schedule will be generated by computer to assign subjects to the treatment sequences. A total of 204 patients will be recruited from seven large-scale gastrointestinal medical centers in China which include Sichuan Cancer Hospital, Chengdu First People’s Hospital, Chengdu Second People’s Hospital, Chengdu Third People’s Hospital, Mianyang People’s Hospital, Mianyang Third People’s Hospital and Langzhong People’s Hospital. This study was approved by the Ethics Committee for Medical Research and New Medical Technology of Sichuan Cancer Hospital (SCCHEC-02-2025-008). The study design is summarized in Figure 1.

### 2.2 Eligibility criteria

The inclusion and exclusion criteria for patients were listed in Table 1.

**TABLE 1:** The inclusion and exclusion criteria for this study.

Inclusion criteria	
1	Male or female aged 18–75 years.
2	Histologically confirmed gastric adenocarcinoma and feasible radical resection for gastric cancer.
3	Eastern Cooperative Oncology Group (ECOG) score of 0 or 1.
4	The main organ function is normal.
5	Informed consent form signed.
Exclusion criteria	
1	Other active malignant tumors within 5 years or at the same time.
2	History of pancreatic disease within half a year.
3	Distant metastasis confirmed by imaging or pathological examination.
4	Allergic to any component of the therapy.
5	Human immunodeficiency virus (HIV) infection.
6	Vulnerable groups, including mentally ill, cognitively impaired, critically ill patients, minors, pregnant or lactating women, illiterate, etc.
7	Severe postoperative complications, including anastomotic fistula, intestinal obstruction, abdominal bleeding, severe infection.
8	Stop taking the experimental medication for more than 2 weeks.

### 2.3 Interventions

After discharge, all enrolled patients will be advised to consume their diet as tolerated, with a focus on eating 5–6 meals per day to avoid gastrointestinal overload. Moreover, patients in PERT group will oral Pancreatin Enteric-coated Capsules (produce by Abbott laboratories GmbH, H20171150), each capsule contains 150 mg pancrelipase microgranules (lipase 10,000 U. Ph. Eur., amylase 8,000 U. Ph. Eur., protease 600 U. Ph. Eur.) which was derived from pig pancreas. The Capsules are commercially available and issued by the project team. According to guidelines (Dominguez-Muñoz et al., 2025; Smith et al., 2016; Gheorghe et al., 2015), we design that the patients after discharge take three capsules (30,000 U) with food three times a day for 1 month in this study. The first participant was enrolled on 6 June 2025.

### 2.4 Outcomes

The primary outcome of the trial is the incidence of gastrointestinal symptoms at 1 month postoperatively in gastric cancer patients. Seven gastrointestinal symptoms (pain, dysphagia, nausea, vomiting, abdominal distension, constipation and diarrhea) are assessed using the MD Anderson Symptom Inventory Gastrointestinal Cancer Module (MDASI-GI) (Wang et al., 2010). We use a 0–10 numerical rating scale (NRS) to assess the severity of symptoms, with 0 being “not present” and 10 being “as bad as you can imagine.” Secondary outcomes include nutritional status and patient compliance. Nutritional status was assessed using the Nutritional Risk Screening 2002 (NRS 2002), Patients were classified as high risk when NRS score was 3 and low risk when NRS score was <3. In addition, hematological markers including lymphocyte count, hemoglobin, serum albumin, and prealbumin also reflected nutritional status, the lower the value, the more severity of malnutrition. Medication adherence in the PERT group is documented through Medication Record Cards.

### 2.5 Follow-up

**FIGURE 2:**
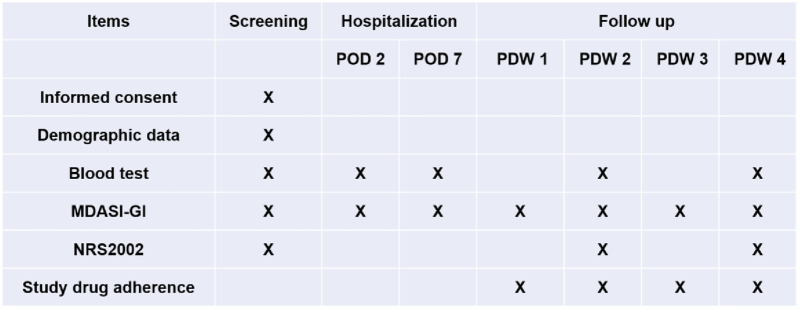
Study schedule. POD, postoperative day; PDW, postdischarge week.

The MDASI-GI and NRS2002 were assessed by patients via Wenjuanxing App. Assessment of MDASI-GI, NRS2002, and blood samples for lymphocyte count, hemoglobin, serum albumin, and prealbumin were collected at baseline before surgery. Follow-up begins postoperatively and continues for 1 month after discharge. Assessment of MDASI-GI occurs at postoperative day 2 (POD 2), postoperative day 7 (POD 7), and twice weekly following discharge. NRS2002 and blood samples are collected once every 2 weeks after discharge (Figure 2). Patients’ self-reported Medication adherence will be recorded. If a patient decides to withdraw, the follow-up will be stopped.

### 2.6 Sample size

The primary outcome of this study is the incidence of gastrointestinal symptoms 1 month after surgery. According to our previous research (Xu et al., 2022), the incidence of gastrointestinal symptoms 1 month after gastric cancer surgery is 50%. The incidence of gastrointestinal symptoms in PERT group is expected to be 30%. The required number of patients has been calculated by PASS V.15.0 software and based on a two-sided significance level of 5%, and a statistical power of 80%. Thus, 204 patients need to be recruited into this study (102 patients in each group) in view of a 10% dropout rate.

### 2.7 Statistical analysis

Continuous variables will be presented as mean ± standard deviation (SD), and categorical variables will be presented as numbers (percentages). The χ^2^ or Fisher’s exact test apply to present the difference of the primary outcome. The t-test is used to demonstrate the statistical difference of the continuous variables such as MDASI-GI score, NRS2002 score and hematological indicators. In addition, the logistic regression (age, sex, nutrition status, style of gastrectomy, PERT will be included as influencing factors) will be used to identify the risk factors affecting the incidence of gastrointestinal symptoms. All randomly assigned patients who receive at least one dose of the study medication will be included in the full analysis set (FAS) according to the intention-to-treat (ITT) principle, and at least one evaluable efficacy outcome is used for the analysis of all efficacy outcomes and baseline characteristics of patients. Patients with study drug adherence of <50% will not be included in the analysis. Statistical significance was defined as a p-value less than 0.05.

## 3 Discussion

As we know, gastrectomy can lead to significant anatomical and physiological changes. Over 90% patients underwent gastrectomy suffered from bloating, diarrhea, and/or weight loss, which might be related to PEI and would affect the quality of life of patients (Cui et al., 2018). The diagnosis of pancreatic exocrine insufficiency (PEI) generally requires a comprehensive assessment integrating clinical symptoms, nutritional status, and pancreatic function. However, symptoms and nutritional markers lack both sensitivity and specificity for PEI. Furthermore, non-invasive tests such as fecal elastase-1 (FE-1) and the 13C-mixed triglyceride breath test, though used to evaluate pancreatic exocrine function, are unreliable in patients following upper gastrointestinal surgery. In addition, many medical institutions do not have access to these tests. In this context, empirical pancreatic enzyme replacement therapy (PERT) has been proposed as a pragmatic clinical approach (Dominguez-Muñoz et al., 2025), which also supports the feasibility of the present trial. Although guidelines recommend using pancreatic enzyme replacement therapy (PERT) to treat PEI, strong evidence for its effectiveness in postoperative gastric cancer remains limited. So far, only three RCTs studies (Armbrecht et al., 1988; Brägelmann et al., 1999; Catarci et al., 2018) exploring PERT after gastrectomy. Armbrecht et al. (1988) showed that PERT could reduce massive steatorrhoea after total gastrectomy based on only 15 patients double-blind crossover trial. Brägelmann et al. (1999) reported patients after total gastrectomy felt better but no significant difference in any symptom. Catarci et al. (2018) found that nutritional status and quality of life could be improved during 3 months after gastric cancer surgery. In order to investigate whether PERT is effective in postoperative gastric cancer, we design this trial and hope to Hope to assist in clinical decision-making.

However, PEI is often ignored by medical staff, due to its the nonspecific nature of the symptoms and the absence of a simple diagnostic test (Croagh et al., 2023). Patient-Reported Outcome (PRO) report can easily make up for this deficiency, which is an important tool to assess the patient’s physical health, functional status, and posttreatment feeling without interpretation bias from the medical personnel (Fiore et al., 2018). We use the MDASI-GI to prioritize symptom burden from the patient perspective, which can better evaluate the effectiveness of PERT.

Furthermore, the PERT-GC01 study protocol enrolls gastric cancer patients who have undergone curative resection, irrespective of prior neoadjuvant therapy. This inclusive design aligns with contemporary clinical practice, where neoadjuvant treatment has become the standard of care. One-month follow-up is short, and will miss long-term outcomes, so further study still be needed.

This protocol establishes a framework for definitively evaluating PERT in gastric cancer patients. Results will inform evidence-based PEI management, with significant implications for survivorship care. By integrating patient-reported outcomes and nutritional metrics, we aim to validate a patient-centered strategy optimizing functional recovery after curative resection.
